# Persistent HIV Viremia: Description of a Cohort of HIV Infected Individuals with ART Failure in Puerto Rico

**DOI:** 10.3390/ijerph13010050

**Published:** 2015-12-22

**Authors:** Gerónimo Maldonado-Martínez, Robert F. Hunter-Mellado, Diana Fernández-Santos, Eddy Ríos-Olivares

**Affiliations:** 1 Data Management and Statistical Research Support Unit, Universidad Central del Caribe, Bayamón 00960, Puerto Rico; diana.fernandez@uccaribe.edu; 2 Internal Medicine Department, Universidad Central del Caribe, Bayamón 00960, Puerto Rico; robert.hunter@uccaribe.edu; 3 Microbiology Department, Universidad Central del Caribe, Bayamón 00960, Puerto Rico; eddy.rios@uccaribe.edu

**Keywords:** HIV, viremia, mean viral load, ART, Puerto Rico

## Abstract

The introduction of antiretroviral therapy (ART) has allowed human immunodeficiency virus (HIV) suppression in patients. We present data of a cohort of Puerto Rican patients with HIV who were under treatment with a steady regime of ART across a time horizon of eleven years. The time periods were categorized into four year stratums: 2000 to 2002; 2003 to 2005; 2006 to 2008 and 2009 to 2011. Socio-demographic profile, HIV risk factors, co-morbid conditions were included as study variables. One year mortality was defined. The *p* value was set at ≤0.05. The cohort consisted of 882 patients with 661 subjects presenting with persistent HIV viral load after a self-reported 12 month history of ART use. In this sub-cohort a higher viral load was seen across time (*p* < 0.05). Illicit drug use, IV drug use, alcohol use, loss of work were associated to having higher viral load means (*p* < 0.05). HIV viral load mean was lower as BMI increased (*p* < 0.001). It is imperative to readdress antiretroviral adherence protocols and further study ART tolerance and compliance.

## 1. Introduction

In the absence of antiretroviral therapy (ART), human immunodeficiency virus (HIV-1) infection causes a longitudinal decline in the CD4 helper T cells with deterioration of the immune system. The lack of effective immunological response increases vulnerability to multiple opportunistic infections [1,2,3,4]. Persistent detectable HIV viral load decreases overall survival and is associated to the detrimental effect of metabolic syndrome and a spectrum of conditions linked to cardiovascular disease [5,6].

The effectiveness of ART in reducing HIV viral load and improvement in CD4 cell counts is well documented [7]. Nevertheless, a major hurdle of the HIV epidemic is the sub-cohort of subjects who continue to have significant detectable viral load despite ART [8,9]. In the setting of clinical trials, ART controls HIV viral load in over 90% of subjects [9]. Laprise *et al.* reported that persistent viral load between 50 and 999 copies/mL was associated to an increased risk of virologic failure [8]. In addition, published data suggests that two or more missed doses of ART are linked to an increased risk of viral failure and death [10]. Ineffective HIV viral response has been attributed to factors such as intermittent access to medications, problems with long term adherence to ART, medication intolerance, adverse effects of the drugs, development of drug resistance virus, lack of continuity in health care, and difficulties with health care access [10]. HIV infection continues to be an important health burden in Puerto Rico. In 2014 a cumulative number of 46,904 persons with HIV were reported, with a 57% mortality (*n* = 26,567) [11,12,13].

In 1992, the Retrovirus Research Center (RRC) initiated a longitudinal HIV registry which continues to recruit subjects. Our HIV ambulatory care facility is a public multidisciplinary clinic that accepts patients with diverse clinical history. We receive newly diagnosed patients, patients who have been previously treated with ART or are currently on therapy with ART from other health care facilities in the island. We also receive subjects who have migrated to the island from other states or countries. The profile of our subjects reveal that they are generally from a lower economic stratum, have drug/alcohol HIV risk practices and often have a lower formal education history. For this study, we selected a cohort of subjects which were new to our ambulatory care clinic and had a one year history of stable ART use. We have compared the group according to presence or absence of detectable HIV viral load and have analyzed the profile of subjects with detectable viral load according to sociodemographic variables, HIV risk factors, presence of comorbid clinical conditions, psychological profile and one year mortality. Considering that protease inhibitors became widely available in the island in 1998 and that we were interested in defining the one year mortality, the time period selected was 1st January 2000 through 31th December 2011.

## 2. Experimental Section

### 2.1. Research Design

We organized our cohort according to year of entry into the ambulatory clinic. The group was stratified in four chronological brackets: January 2000 to December 2002; January 2003 to December 2005; January 2006 to December 2008; January 2009 to December 2011. The dependent variable was detectable viral load defined as greater than fifty (50) copies/mL. Thus the inclusion criteria were newly seen subjects in our ambulatory care clinic and a self-reported twelve (12) month steady use of antiretroviral therapies (ART). Steady use of ART was defined as use of at least three antiretroviral drugs in a daily basis. Additional analyses were made according to the presence or absence of detectable HIV viral load.

A total of 882 new subjects with a 12 month self reported history of ART were seen in the HIV ambulatory care clinic in the defined period. A subcohort of 661 patients had detectable HIV viral load. After written informed consent, an initial questionnaire was completed via interview, the medical record was abstracted and data tabulated into our data base. The data abstraction protocol included past medical data from twelve (12) months prior to the initial interview.

Study variables were gender, age, body mass index, risk behavior variables and co-morbid conditions. We established the one year mortality amongst the different groups in our cohort. The body mass index (BMI) at study entry was calculated using the following formula $\mathrm{BMI}\;=\;\frac{\mathrm{mass}(\mathrm{lb})}{{\mathrm{height}}^{2}}*\;703$, then categorized into three groups, as follows: underweight (BMI ≤ 18.4–18.49), normal (BMI 18.5–24.99) and overweight/obese (BMI > 25). The alcohol abuse variable was defined as: consuming an average of more than two drinks per day or more than 14 drinks per week or Alcoholics Anonymous attendance in the last 12 months. The variables in the psychological profile were history of mental disorders (schizophrenia, bipolar state, psychosis and anxiety), isolation, antisocial behavior, loss of work. Co-morbid conditions included diabetes mellitus, renal insufficiency and cardiovascular disease (hypertension, heart failure, ischemic events and vascular disorders). Risk behavior variables were intravenous (IV) and illicit drug use.

### 2.2. Statistical Analyses

The Statistical Package of Social Sciences program version 21.0.1 (IBM-SPSS, Chicago, IL, USA) was used to perform univariate, bivariate and multivariate analyses. Descriptive analyses included frequencies, percentages, central tendency measures (mean and median) and dispersion measures (standard deviation measures and quartiles). A normality test using the Shapiro-Wilk estimate was performed in all variables in order to select the correct parametric or non-parametric test. Differences between mean viral load and year bracket were analyzed with an ordinary one-way ANOVA with a Bonferroni correction. Differences among proportions were assessed using a chi-square test approach or Fisher’s exact test with a linear-by-linear correction.

The general linear model (GLM) is a statistical linear model derived from the multiple regression model: Y = XB + U; where *Y* is a matrix with series of *p* multivariate measurements; X is a design matrix; B is a parameter matrix and U is the error/noise matrix. In this study, a GLM mixed design ANOVA was built in order to assess the change of estimated marginal mean viral load through a time trend within groups, controlled by use of antiretroviral therapy. Inclusion of factors into the model was mediated by having ≤0.05 at a bivariate level. A Box’s M test was performed to assess if our models have the assumption of equality of covariance homogeneity (*p* > 0.05). The Pillai’s Trace estimator was used in order to know if the fixed factors contribute in the variability of the dependent variable (*p* ≤ 0.05).

A test of between-subjects effect with a pairwise comparison using a Bonferroni adjustment for multiple comparisons was applied to perceive statistical differences between and within the groups. The significance level (α) was set at ≤0.05.

## 3. Results and Discussion

### 3.1. Bivariate Profile

**Figure 1:**
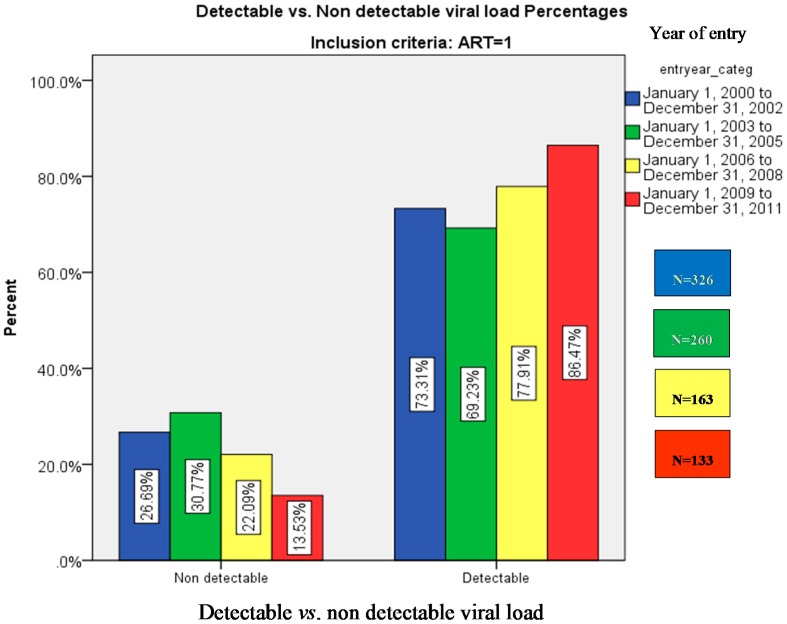
Detectable *vs.* non-detectable viral load (% patients).

There were 661 (75%) patients with steady ART use and detectable HIV viral load and 221 (25%) subjects without detectable viral load. Figure 1 presents the percentage distribution of subjects with detectable vs. non detectable viral load across the four periods. In Table 1 we present a bivariate analysis comparing the profile of subjects with and without detectable HIV viral load. The use of IV drugs, cannabis use, the presence of diabetes mellitus and alcohol abuse were significantly associated to having a detectable viral load (*p* < 0.05). There was no difference in the one year mortality between groups. In Table 2, the mean value of detectable viral load in subjects was analyzed across the time. Values with zero detectable viral loads were excluded. The mean viral load was significantly higher in the last time period (2009–2011) (*p* < 0.0001). Bonferroni *post hoc* test demonstrated that all three time periods significantly differ from the initial one (2000–2002).

**Table 1:** Detectable and non-detectable viral load after steady ART use with sociodemographic, risk factors, co-morbid criteria, psychological profile and one year mortality. Puerto Rico, 2000–2011.

Variables *n* (%)	Non Detectable Viral Load ^&^ *N* = 221	Detectable Viral Load ^&^ *N* = 661	*p* ^a^
**Sex**			
Male	133 (60.2)	409 (61.9)	>0.05
Female	88 (39.8)	252 (38.1)	>0.05
**Body Mass Index**			
Underweight	14 (6.8)	58 (9.6)	>0.05
Normal	91 (44.2)	287 (47.4)	>0.05
Overweight/Obese	101 (49)	261 (43.1)	>0.05
**Risk factors** ^^^			
Intravenous drug use	57 (25.8)	212 (32.1)	<0.05
Heroin use	78 (35.3)	230 (34.8)	>0.05
Cocaine use	110 (49.8)	334 (50.5)	>0.05
Heroine + Cocaine use	76 (34.4)	215 (32.5)	>0.05
Amphetamine use	55 (25)	145 (22)	>0.05
Crack use	165 (25.3)	161 (24.4)	>0.05
Cannabis use	90 (40.7)	319 (48.3)	<0.05
**Co morbid conditions **^^^			
Diabetes mellitus	13 (5.9)	55 (8.4)	<0.05 ^b^
Renal insufficiency	4 (1.8)	27 (4.1)	>0.05 ^b^
Cardiovascular disease	37 (16.8)	95 (14.4)	>0.05 ^b^
**Psychological profile** ^^^			
Mental disorders	9 (4.1)	35 (5.3)	>0.05 ^b^
Isolation	93 (42.1)	253 (38.3)	>0.05
Antisocial behavior	78 (35.5)	259 (39.2)	>0.05
Loss of work	43 (19.5)	104 (15.7)	>0.05
Alcohol abuse	37 (27.2)	107 (30.8)	<0.05
**Mortality profile**			
One year mortality	11 (5.0)	41 (6.2)	>0.05 ^b^

**^&^** Subjects in steady antiretroviral therapy; ^^^ Not mutually excluded data; ^a^ Chi-square test for independence (Linear by Linear = 8.780; *p* < 0.05); ^b^ Fisher’s exact test.

**Table 2:** Viral load mean value across time in subjects with detectable viral load only, Puerto Rico, 2000–2011.

Strata *	*n*	$\bar{x}$	95% Confidence Interval for Mean		*p*
			Lower Bound	Upper Bound	
1 January 2000 to 31 December 2002	239	122,712.97	97,215.61	148,210.33	<0.0001
1 January 2003 to 31 December 2005	180	144,584.36	119,126.10	170,042.63	
1 January 2006 to 31 December 2008	127	180,567.81	120,302.42	240,833.20	
1 January 2009 to 31 December 2011	115	191,196.14	105,837.38	276,554.90	
Total	661	151,699.32	129,738.96	173,659.69	

^*^ Bonferroni *post hoc* test *p* < 0.05.

### 3.2. Multivariate Profile

In Table 3 we present the mixed model ANOVA. The model proved to be appropriate (Box’s M = 44.54; *p* > 0.005). All fixed factors contributes to the model (Pillai’s trace F = 16.332; *p* < 0.0001). We use estimated marginal means (EMM) for the expression of the HIV viral load. We were interested in defining variables relevant in explaining the incremental values of HIV viral load across time. For the model, we included variables reported in the literature to be relevant for patients with high HIV viral load in subjects in the context of ART use. A significant association with higher HIV viral load and all the selected variables were seen. In the group of males a significant steady increase mean viral load was seen (*p* < 0.001). When examined independently all levels of BMI were associated to increased HIV viral load, although a low BMI was associated with the steepest increase in detectable viral load across time. Intravenous drug use, cocaine, crack and cannabis use were also associated with an increase in mean viral load (*p* < 0.05). Alcohol abuse and loss of work were seen to be associated to having higher viral load means across time (*p* < 0.05). Our analysis suggests that the increment of HIV viral load across the time had a stronger association with the selected variables in the last period examined. The group of factors (male gender, low BMI, IV and illicit drug use, alcohol abuse) which have been traditionally associated to ART failure, appears to be present in a higher frequency in the last period studied. We have identified loss of work as an additional factor associated to ART failure.

**Table 3:** Estimated marginal means across time (linear trend design) in subjects with detectable viral load.

Variables ^a^	2000–2002	2003–2005	2006–2008	2009–2011	*p*
**Sex**					
Male	139,549.46	141,812.47	161,479.31	196,673.59	<0.0001
Female	94,336.87	149,859.90	202,568.12	181,675.81	<0.0001
**Body Mass Index**					
Underweight **^b^**	103,463.25	142,623.17	281,912.50	462,419.88	<0.05
Normal **^b^**	142,383.22	166,449.46	200,297.44	224,387.67	<0.05
Overweight/Obese **^b^**	107,025.76	115,098.19	126,806.57	135,78.50	<0.05
**Risk factors**					
Intravenous drug use	119,838.63	156,827.40	206,664.17	239,627.09	<0.05
Cocaine use	121,578.97	172,129.96	196,431.25	208,812.54	<0.05
Crack use	131,924.75	165,193.00	186,316.92	204,891.33	<0.0001
Cannabis use	106,862.19	178,471.37	212,686.60	154,391.48	<0.05
Loss of work	111,226.72	149,996.45	161,694.90	196,901.00	<0.05
Alcohol abuse	122,813.22	140,426.00	186,923.75	230,248.68	<0.05

**^a^** Pillai’s trace F = 16.33; *p* < 0.0001; **^b^** Bonferroni adjustment for multiple comparisons.

### 3.3. Discussion

The majority of newly registered subjects in our cohort have detectable HIV levels in spite of prescribed ART. We believe this is a reflection of the particular HIV risk profile of patients who receive health care in our ambulatory clinic. Our center is one of the few facilities in the island which are government sponsored and predominantly serve a population with government sponsored health care insurance [14,15]. The risk profile of subjects who receive care in our clinic not only have a higher prevalence of IV drug use, illicit drug consumption and alcohol abuse, but many subjects are known to concurrently practice all high risk behaviors [9]. Evaluating the mediating and possibly synergistic effects of concurrent high risk practices with persistently detectable HIV viral load is a complex endeavor, however our group and others have implicated that IV drug use as a significant factor associated to virological failure in subjects with persistent ART use. Other studies have suggested that concurrent or tandem HIV risk practices as well as older age and male gender are detrimental towards control of HIV viral load [15].

The problem of medication adherence and toxicity is a serious issue in our cohort. We have demonstrated that the combination of risky behavior and being underweight resulted is a detrimental scenario that may contribute to ART inefficacy. Previous studies have also linked the association of viremic suppression failure, IV drug use and alcohol abuse with lack of medication adherence [15,16]. We have hypothesized that ineffective ART use in the presence of certain HIV risk behaviors continue to have a relevant association with detectable HIV viral load across time. The presence of loss of work is a new social factor associated to HIV viremia with ART use.

We demonstrate that in a time horizon of eleven years, the risk scenarios associated to detectable HIV viral load, have increased in prevalence in the population of patients we serve. Similar to our findings, other studies suggest that illicit drug use, a low socio demographic profile and high rate of abnormal psychological profile are factors which correlate with ART failure [16]. Issues of non-adherence, adverse effects of the medications along with other comorbid conditions may be likely responsible for the HIV viral load incremental blips and possibly contributing to the effectiveness of ART [17].

We were interested in comparing variations across time of detectable viral load within each gender group. Our study reveals that within males the proportion with detectable viral loads has been progressive increasing. In the last period examined, a large percent of males who entered the cohort had detectable viral levels. In contrast, although females who present with detectable viral load represent more than half percent of the cohort, the rate with detectable virus tend to stabilize in the last time bracket of the eleven years study period [18,19]. In our population the risk factor of illicit drug use is much more common in males than in females, arguing in favor of gender representing a surrogate marker for certain risk practices [20].

Efforts to restructure the medication adherence protocols, along with introduction of innovative methods to improve medication adherence are essential for our community. This issue has been identified in other communities leading to innovative interventions [21]. Our study has identified that the use of illicit drugs and alcohol abuse as the important risk practice, with loss of work and presence of diabetes mellitus as relevant factors to the presence of detectable viral load in ART pretreated subjects.

Access to the necessary ART drugs is not an important issue in our center since all medications prescribed to the subjects are offered free of charge to the patients. Nevertheless adverse drug effects and the issue of toxicity are likely important factors in our patients since a substantial number of subjects have comorbidities in the form of diabetes mellitus and cardiovascular diseases [22]. Our data is not able to discern if these conditions arise as a consequence of ART or were dependent on other factors such as diet, family history, co morbid infections such as endocarditis, or viral infections. In addition, possible changes in the absorption or pharmacodynamics of individual ART drugs in patients with low body mass index and presence of certain co-morbidities have been described [23,24]. Our data suggest that these variables are not mutually exclusive.

The finding that the presence of detectable HIV viral load is not associated to increased 1 year mortality has been described in other studies [25]. We selected one year as the terminal point in order to include the entire cohort in our study. Nevertheless changes in long term mortality in patients with detectable HIV viral load are likely to be present with longer follow up. It may also suggest that some patients are rescued from progressive immune deterioration resulting in improvement in survival. We plan further studies to address this point.

Other studies have also suggested that underweight subjects in the high risk category for ART failure, are partly responsible for lack of control of the HIV epidemic in spite of improvement in ART therapy [26]. In addition, poor HIV health education component may be a strong cofactor in explaining a possible poor adherence issue. Variables of a sociodemographic and behavioral nature continue to present hurdles in our group of subjects. Attempts to slow down the growth of the epidemic have focused on the early detection of subjects followed by ART initiation [27]. Our data suggests that strategies to optimize effectiveness of ART such as robust follow-ups, daily pill count strategies and frequent pill diaries needs to be readdressed.

## 4. Conclusions

The subjects in our cohort are not representative of the HIV infected population at large; however they remain a relevant and important cohort in the community of infected patients, which will continue to defeat the efforts to control the rate of new HIV infections. Present prevention and intervention strategies must be reevaluated and readdress in order to redirect the efforts of stopping the HIV epidemic. Further intervention protocols will need to consider the cohort of subjects with the high HIV risk practices associated to ART failure.
